# An Introduction to Bioinformatics for Glycomics Research

**DOI:** 10.1371/journal.pcbi.1000075

**Published:** 2008-05-30

**Authors:** Kiyoko F. Aoki-Kinoshita

**Affiliations:** Department of Bioinformatics, Soka University, Tokyo, Japan; Whitehead Institute, United States of America

## Introduction

Carbohydrates are considered the third class of information-encoding biological macromolecules. “Glycomics,” the scientific attempt to characterize and study carbohydrates, is a rapidly emerging branch of science, for which informatics is just beginning. Glycomics requires sophisticated algorithmic approaches. Several algorithms and models have been developed for glycobiology research in the past several years. This tutorial will provide a brief introduction to the field of glycome informatics, which will include a primer on glycobiology as well as descriptions of the algorithms and models that have been developed in this field.

The four essential molecular building blocks of cells are nucleic acids, proteins, lipids, and carbohydrates, often referred to as glycans. Nucleotide and protein sequences are at the heart of nearly all bioinformatics applications and research, whereas glycan and lipid structures have been widely neglected in bioinformatics. However, glycans are the most abundant and structurally diverse biopolymers formed in nature. Bound to proteins, as glycoproteins, they are known to affect the functions of proteins. More than half of all protein sequences deposited in the SWISS-PROT databank include potential glycosylation sites and thus may be glycoproteins. Based on an analysis of well-annotated and characterized glycoproteins in SWISS-PROT, it was concluded that more than half of all proteins are glycosylated [1].

The development and use of informatics tools and databases for glycobiology and glycomics research has increased considerably in recent years. However, the general development in this field can still be considered as being in its infancy when compared to the genomics and proteomics areas. In terms of bioinformatics in glycobiology, there are several paths of research that are currently in progress. The development of algorithms to reliably support the characterization of glycan structures for high-throughput applications is the most immediate demand of the glycomics community. Additionally, several major glyco-related projects (Consortium for Functional Glycomics [2], KEGG Glycan [3], GLYCOSCIENCES.de [4]) are maturing and provide well-structured glyco-related data that are awaiting data mining and analysis. With the exciting new developments in carbohydrate arrays and automated MS annotation, the analysis of the glycome has reached a new level of sophistication, which requires broader informatics support. This tutorial aims to give an overview of the current status of carbohydrate databases, the newest analytical techniques, as well as the informatics needed for rapid progress in glycomics research.

## Background

Complex carbohydrates are chains of monosaccharides, often called glycans, and are often found attached to proteins (to form glycoproteins) and lipids (glycolipids, glycosphingolipids, etc.). Glycoproteins are usually on the cell surface, where they are recognized by bacteria, viruses, and other proteins, such as lectins, in order to facilitate various crucial functions. It is also known that glycans are involved in a variety of biological processes including protein folding and signalling events.

The complex structure of glycans has been a bottleneck in the structure determination and thus data accumulation of glycan structures. This is confounded by the complex biosynthetic pathways of glycans. It is known that glycan-specific diseases called CDGs (congenital disorders of glycosylation) are caused by defects in these pathways [5]. Furthermore, there have been many reports on glycan markers related to human diseases such as cancer and autoimmune diseases [6],[7].

### 

#### Carbohydrate Structure Notation

**Figure 1:**
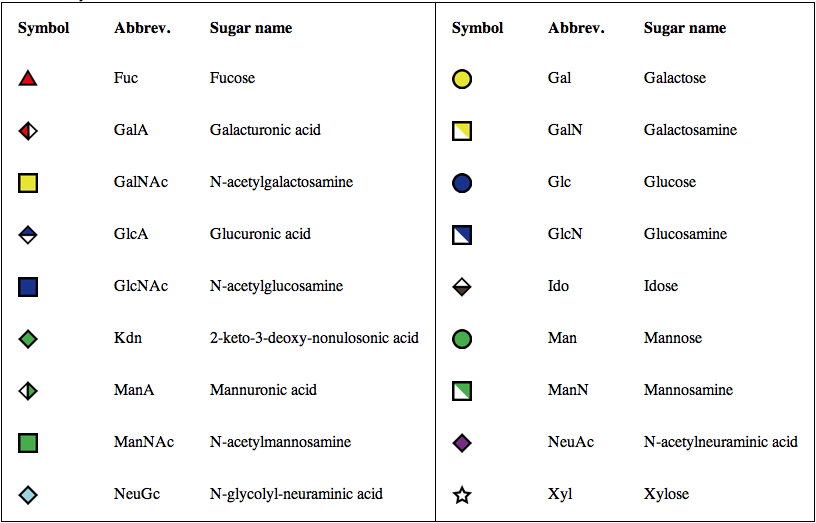
Standard representation of carbohydrate chains as proposed by the Consortium for Functional Glycomics.

Complex carbohydrates are composed of monosaccharides that are covalently linked by glycosidic bonds, either in the α or β form. Unlike DNA and proteins, however, monosaccharides may be linked to one or more other monosaccharides, such that they form a branched tree structure. In order to formulate a standardized notation for glycans, the Consortium for Functional Glycomics (CFG) proposed a standard symbolic representation for those monosaccharides that are found most in nature, which has been employed in [8]. This representation (as given in Figure 1) will be utilized throughout this tutorial.

**Figure 2:**
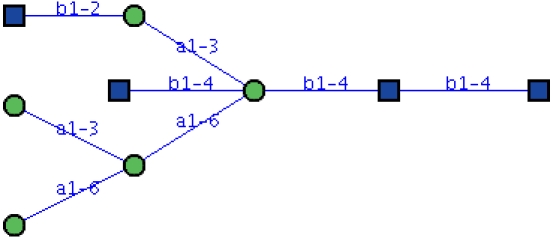
An example of an N-linked glycan, illustrated as a tree structure rooted at the right side and branching toward the left.

Carbohydrates are most classically drawn as a tree in a two-dimensional plane, with the root monosaccharide placed at the right-most position and children branching out toward the left. Each node represents a monosaccharide, and each edge represents a glycosidic linkage, which includes the carbon numbers that are bound and the conformation. An example of an N-linked glycan is given in Figure 2.

Although the two-dimensional notation is nice and pretty, it is not suitable for storage in a database, let alone for bioinformatic analysis. The IUPAC–IUBMB (International Union of Pure and Applied Chemistry–International Union of Biochemistry and Molecular Biology) has specified the “Nomenclature of Carbohydrates” to uniquely describe complex oligosaccharides based on a three-letter code to represent monosaccharides (e.g., “gal” for galactose and “man” for mannose). Each monosaccharide code is preceded by the anomeric descriptor and the configuration symbol. The ring size is indicated by an italic *f* for furanose or *p* for pyranose. The carbon numbers that link the two monosaccharide units are given in parentheses between the symbols separated by an arrow. For example, the structure in Figure 2 would be represented as: β-D-Glc*p*NAc-(1→4)-[β-D-Glc*p*NAc-(1→2)-α-D-Man*p*-(1→3)][α-D-Man*p*-(1→3)-[α-D-Man*p*-(1→6)]-α-D-Man-(1→6)]-β-D-Man*p*-(1→4)-β-D-Glc*p*NAc-(1→4)-β-D-Glc*p*NAc. In such a way, long carbohydrate sequences can be adequately described in abbreviated form using a sequence of letters.

However, as we discuss in the next section, it is not always possible to obtain a full and exact representation of carbohydrates due to the difficulties in sequencing them. Currently, the most popular method for complex carbohydrate sequencing is mass spectroscopy (MS). However, this process is often incomplete and error-prone. For example, unless one uses MS in tandem it is nearly impossible to distinguish between isomeric monosaccharides (e.g., glucose, galactose, and mannose are all hexoses with the same mass). As any spectrometrist will state, MS in tandem is a rather tedious process, even for one carbohydrate structure. Thus, for those developing databases, the notation for carbohydrates must be flexible enough to capture all the data at hand but also be able to account for ambiguities.

There are currently in use several different notations for carbohydrates, which developed out of the construction of some major databases during a time when no standard notation for carbohydrates existed. Briefly, these notations are KEGG Chemical Function (KCF) format, which represents glycans using a connected graph, LINUCS (Linear Notation for Unique Description of Carbohydrate Sequences), which provides a unique and linear notation for glycans, and Linear Code by GlycoMinds, which provides a commercial complex carbohydrate database [9].

## Databases

As of the time of this writing, there are three major databases for complex carbohydrates, Glycosciences.de, KEGG GLYCAN, and the database developed by the Consortium for Functional Glycomics (CFG). All three databases are based on the CarbBank database developed in the 1990s by the Complex Carbohydrate Research Center (CCRC) at the University of Georgia [10]. These databases have been summarized in Table 1.

**Table 1:** The Three Major Publicly Available Carbohydrate Databases Are Listed Along with the URLs and Literary References.

Database Name	Description	URL	Reference
Glycosciences.de	Database of glycan structures and mass spectral data, based at the German Cancer Research Center	http://www.glycosciences.de	[4]
KEGG GLYCAN	A part of the KEGG database containing glycan structures extracted from CarbBank and subsequently linked with the GENES and PATHWAY information in KEGG. Glycosyltransferases and glycan binding protein data have also been organized in KEGG BRITE	http://www.genome.jp/kegg/glycan/	[3]
CFG	Developed by the Bioinformatics Core of the CFG, this database contains structures from CarbBank and a seed database provided by GlycoMinds. They have been subsequently linked with tissue and cell data, glycan array information, and glycans specifically synthesized by the CFG.	http://www.functionalglycomics.org/	[2]

The major issue that was facing the glyco-informatics community was the fact that each of these databases represented their glycan structures in different formats. Glycosciencse.de uses the LINUCS format, KEGG the KEGG Chemical Function (KCF) format, and CFG the IUPAC format. In September 2006, a workshop was held at the National Institutes of Health (NIH), United States, where glycobiologists and glyco-informaticians gathered to discuss a standard exchange format for carbohydrate structures. At this meeting, the GLYDE-II XML format for glycans and glycoconjugates, developed by the CCRC, was agreed upon as the standard format for exchanging carbohydrate data [11].

## Glycome Informatics Methods

Along with the development of these glycan databases over the past few years, bioinformatic methods for analyzing glycan structures have also appeared. In general, these can be classified into the following six categories: glycosylation analysis, glycomics, glycan biomarker prediction, glycan structure analysis, glyco-gene expression analysis, and glycan structure mining.

In the area of research in the first three categories of glycosylation analysis, glycomics and glycan biomarker prediction may be of most interest to biologists, whereas the latter are (currently) active areas of research in the informatics community. Thus, the literature is rich in research in the former areas, and it is hoped that the latter areas will be able to develop and produce more interesting results as these technologies advance. In any case, these areas are all covered equally in this section.

### 

#### Glycosylation Analysis

Since the methods in this section have been summarized nicely in two previous reviews [12],[13], they are only briefly mentioned for reference.

#### *Prediction of glycosylation binding sites on proteins*

As one form of post-translational modification, glycosylation affects the function of the modified protein. Thus, many methods have been developed to predict glycosylation sites based on the amino acid sequence. These methods have been summarized in Table 2.

**Table 2:** Glycosylation Prediction Programs.

Name	Description	URL
Big-PIPredictor [41]	GPI-anchor prediction	http://mendel.imp.univie.ac.at/sat/gpi/gpi_server.html
GlyProt [42]	In-silico glycosylation	http://www.glycosciences.de/modeling/glyprot/
GlySeq [14]	Statistical analysis of glycosylation sites	http://www.glycosciences.de/tools/glyseq/
GPI-SOM [43]	Identification of GPI-anchor signals using a Self Organizing Map (SOM)	http://gpi.unibe.ch
NetNGlyc [44] and NetOGlyc [45]	N- and O-glycosylation prediction; also available as SOAP-based web services	http://www.cbs.dtu.dk/services/NetNGlyc/ and http://www.cbs.dtu.dk/services/NetOGlyc/
NetCGlyc [46]	C-mannosylation site prediction from mammalian proteins	http://www.cbs.dtu.dk/services/NetCGlyc/
YinOYang [44]	Neural network predictions for O-β-GlcNAc binding sites in eukaryotic proteins, using predicted phosphorylation sites	http://www.cbs.dtu.dk/services/YinOYang/

#### *Statistical analysis of amino acids surrounding the glycosylation binding site of a glycoprotein*

The statistical analysis of amino acids surrounding glycosylation binding sites has been an active area of research by the German Cancer Research Center. One of their tools called GlySeq [14] statistically analyzes the amino acids surrounding the glycosylation sites based on protein sequences from Swiss-Prot and the Protein Data Bank (PDB). These statistics are publicly available in the GlySeqDB database.

In addition to analyzing the surround sequence, a tool called GlyVicinity performs a statistical analysis of a PDB entry by computing the frequency of amino acids within a user-definable distance up to 10 Å of carbohydrate residues. This tool performs on top of the data in GlyVicinityDB, which contains distance information of the amino acids in the spatial vicinity of carbohydrate residues in PDB entries [14].

#### *Mathematical modeling of glycosylation*

In other work at Johns Hopkins University, a model to mathematically formulate N-glycosylation was developed [15] based on a previous model that formulated the initial stages of N-glycosylation up to the first galactosylation of an oligosaccharide [16]. This new model characterizes the substrate specificities of known glycosyltransferases as a rule table. Thus, given a set of expressed genes, the list of possible glycans synthesized by the input can be predicted. This model was further enhanced to incorporate enzyme kinetics such that concentrations of structures could be computed using nonlinear algebra. The results were supported by experimental evidence.

#### Glycomics (Mass Analytics)

The field of glycomics can be defined as the technology to determine carbohydrate sequences (structures) using mass spectral data. This area of research has been the most desired by the glycobiology community due to the tedious process traditionally being used to characterize glycans and glycoproteins. In particular, each mass peak was manually annotated by experts, resulting in months of analysis for one mass spectrum.

This problem was conventionally solved by developing a database of theoretical mass spectra corresponding to known glycan structures. Thus newly produced MS data could be compared with the theoretical spectra to find the most similar one, thus providing a clue as to the structures behind the new spectra [17].

More recently, as a result of the large volumes of MS data being produced by the CFG, the Cartoonist program was developed to automatically annotate N-glycans in MALDI-MS data [18]. The Cartoonist labels peaks in MALDI spectra of permethylated N-glycans with diagrams, or cartoons, of the most plausible glycans consistent with the peak masses and the types of glycans being analyzed. There are three main parts to Cartoonist: (i) select annotations from a library of biosynthetically plausible cartoons, (ii) determine the precision and calibration of the machine used to generate the spectrum automatically based on the spectrum itself, and (iii) assign a confidence score to each annotation. As a result, the Cartoonist provides a list of all plausible annotations for each peak, associating each annotation with a confidence score.

In an attempt to predict any type of glycan structure from mass spectra, the GLYCH method was developed to use a dynamic programming method and a listing of all possible fragment types of glycans [19]. There are still difficulties, however, in distinguishing between different branches. Other online tools for annotating glycan structures from mass peaks include GlycoPep ID [20], GlycoMod [21], and GlycoPeakFinder [22].

#### Glycan Biomarker Prediction

Many glycan motifs are known to be involved in a variety of diseases including cancer [23]. Thus it came about that methods to predict characteristic glycan substructures from sets of known glycans may be useful in predicting such biomarkers. From the bioinformatics side, kernels are well-known as useful classifiers for large sets of data given a vector of features from which to extract the most likely candidates. Thus, several kernel methods for glycan biomarker prediction and classification have been developed. For an introduction to kernel methods, the interested reader is referred to the book *Learning with Kernels* by Scholkopf and Smola [24]. Support vector machines (SVMs) are the most popular kernel method, where two (or more) classes of objects can be trained such that new objects can be classified according to the trained features of the objects. In addition to training and classification, new methods for “feature extraction” have been utilized in SVMs such that the most relevant features to the classification problem can be identified to improve training. This feature extraction method has subsequently been used, as will be described here, to extract possible glycan features that may serve as biomarkers. More details on feature extraction for computational biology can be found in the literature [25].

In glycome informatics, the layered-trimer kernel was first developed and used to verify the utility of using kernels for glycan biomarker prediction [26]. This method was further expanded as the *q*-gram distribution kernel [27], and a separate method combining multiple kernels was later used for glycan structure classification [28].

#### *Layered-trimer kernel*

Taking advantage of the fact that the glycan substructures at the leaves are more prone to be recognized compared to the root structures attached to proteins, a weighting scheme was employed that differentiated substructures based on their “depth” or the “layer” of the substructure, the number of glycosidic linkages between the substructure and the root. Furthermore, it is known that glycosyltransferases interact with three monosaccharides on average. Thus, glycan structures were decomposed into trimers. This produced a feature vector of trimers distinguished by layer, which was tested using a dataset of glycans related to different blood components as well as to leukemic cells. These annotations were retrieved from the original CarbBank database.

The kernel was defined using a weighting parameter for the layer of each glycan substructure, according to the following equation. Given the feature vectors for two glycans *X* and *Y*, their inner product is calculated as Σ*w_k_x_k_y_k_*, where *k* is a feature, and so the summation is taken over all features. The weighting parameter *w_k_* is set to 1 when the layer of feature *k* is 1. Otherwise, *w_k_* = *1*−*exp*(−α*h*), where α is a positive constant to weight *h*, the layer of the matching substructures.

Using this kernel on the leukemia dataset described above, the model was able to extract a feature that was highly characteristic of leukemia, which was corroborated by experimental evidence.

#### Q*-gram distribution kernel*

This method extended the layered-trimer kernel in order to account for potential glycan biomarkers that were smaller or larger than trimers, without the use of layers, since it was assumed that layer information could be subsumed by the wider distribution of features. As a result, the *q*-gram distribution kernel could predict leukemia markers as equally well as the previous model, and, in addition, it found that sulfation was a major marker for cystic fibrosis, which is smaller than a trimer. Thus, a more flexible kernel was developed.

#### *Multiple kernel*

Finally, to more efficiently handle the large number of features required by the *q*-gram distribution kernel, a hierarchical model was developed, where a kernel for each *q* was first developed, upon which another kernel was trained to extract the best feature from the best kernel. This model was again shown to produce similar results to the original layered-trimer kernel.

#### Glycan Structure Analysis

The tree structure of glycans has been a topic of interest especially for bioinformaticians interested in trees. Traditionally, RNA structures and phylogenetic analyses have been the focus of tree-based algorithms. However, these structures result in trees with information at the leaves, with internal nodes representing relationships between the leaves. Thus, glycans have provided a structure where internal and external nodes all represent the same type of object: monosaccharides. As a result, glycan structure alignment using tree alignment algorithms and glycosidic linkage score matrices has been developed and analyzed.

#### *Glycan structure alignment*

**Figure 3:**
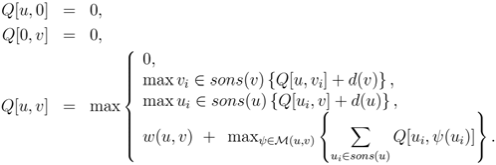
Dynamic programming algorithm for aligning two tree structures, where sons(*x*) refers to the children of node *x*, *d(x)* is a gap penalty, and *M(u,v)* refers to all mappings between the children of nodes *u* and *v*.

The first application of tree-structure alignment using dynamic programming applied to glycans was the algorithm called KEGG Carbohydrate Matcher, or KCaM [29]. By comparing two nodes between two trees based on the mapping of the respective children, the dynamic programming algorithm in Figure 3 can be used to align two glycans. Here, *M(u,v)* is the mapping between the children of *u* and *v*, and *sons(x)* is the set of children of node *x*, and *w(u,v)* is the similarity score between nodes *u* and *v*, which can be defined by a weighting between the matches of the monosaccharide type and the glycosidic linkage between the monosaccharide and its parent (which is null at the root). Considering the fact that gaps really are not expected to appear often in meaningful glycan structure alignments, the gap penalty *d* may be set to a very large value to penalize gaps more heavily.

#### *Glycan substitution matrix*

This algorithm may now be used to analyze monosaccharide similarity, as in amino acid similarity, as represented by amino acid substitution matrices such as PAM [30] and BLOSUM [31]. However, unlike proteins, there are not functionally distinguished families of glycans, as they are considered more as modifiers of protein functions as opposed to function-regulating molecules in and of themselves. Furthermore, the linkage conformation information should also be taken into consideration. Therefore, an appropriate glycan score matrix would be one where glycosidic linkages and the monosaccharides being linked should be used as the basic unit for comparison. Glycan families can be defined computationally or be generated based on the classic classification of glycans, which is derived from the core structure, determined by the conjugate to which the glycans are bound.

Once the appropriate classes of glycans are defined, the KCaM alignment results can be used to calculate the frequency of alignment of glycosidic linkages, which includes the full linkage information (carbon numbers and conformation), as well as the two monosaccharide names which are linked (hereafter called “links”). This score matrix of links is thus the log odds score of the expected frequency of alignment of link pairs [32]. From this matrix, we expect to find those links that are positioned similarly, and thus those that are potentially “functionally” similar. This matrix can also be used to improve the KCaM algorithm to produce more biologically meaningful results.

#### Glyco-Gene Expression Analysis

In an attempt to overcome one of the major issues in glycomics, glycan structure characterization through MS, a bioinformatic method to predict glycan structures in a particular cell through the gene expression profiles was developed [33]. In this method, the concept of a “co-occurrence score” was calculated based on the co-occurrence of pairs of links within the same glycan structures. It was expected that by doing so the substrate specificity of glycosyltransferases could be captured in a single numerical matrix. Once this co-occurrence score matrix was developed, it could be used to make predictions from expression data.

**Figure 4:**
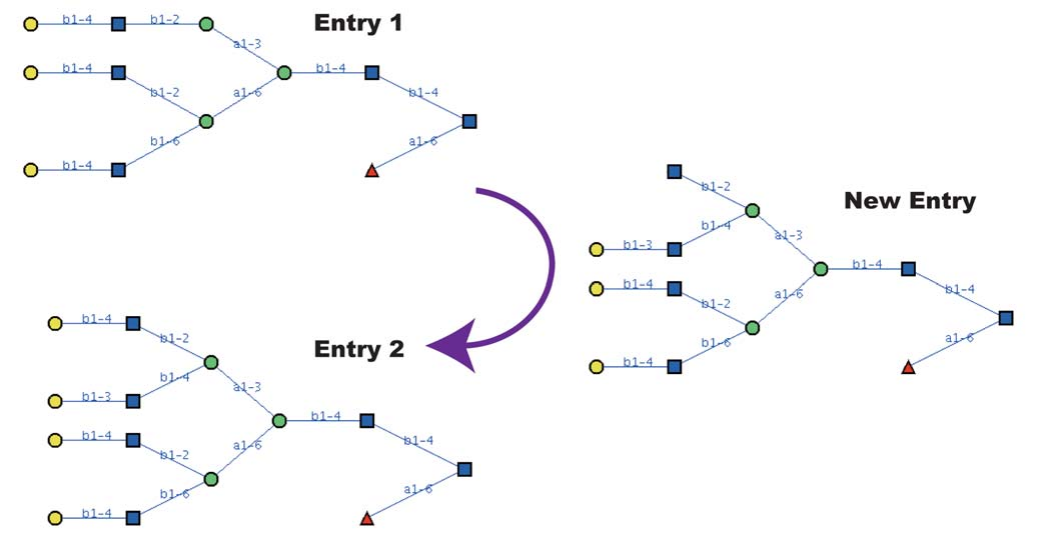
An example of the generation of a new glycan entry given two similar glycans. Since Entry 2 contains just two more nodes than Entry 1, and since in almost all cases glycosidic linkages are synthesized one by one, we can assume that the New Entry exists and can be added as a new structure.

This method was further improved such that (i) the database of glycans were augmented with new glycans that should exist and (ii) the prediction score for glycans used the expression values directly as opposed to using binary values. The first step was performed by analyzing the database of glycans and finding those that differed by more than one link. That is, considering the fact that glycosyltransferases typically catalyze only one link at a time, if two similar glycans in the database existed, but differed by say two to four links, then “intermediate” glycans that should be catalyzed in the process of synthesizing the larger structure should also exist, and these “intermediate” glycans are added to the database. Figure 4 is an example, where a new entry can be presumed to exist and thus added to the database based on the two existing entries Entry 1 and 2. With this augmented database, it is hoped that better scoring results will be obtained. As a result, using a dataset of acute lymphocytic and myelocytic leukemia, those structures containing Lewis-a, Lewis-x, or sialyl-Lewis-x epitopes, which are known to be related to cancer, were often ranked more highly compared to the original method. Furthermore, the newly added glycan entries were also found to be ranked highly in the results [34].

#### Glycan Structure Mining

Lectins are known to recognize specific glycan structures, whose binding events trigger signalling processes to occur. However, oftentimes the specific structures being recognized are unknown. For example, siglecs are suspected to recognize patterns not only at the leaves of glycans but also further deeper in the chain [35]. In order to find such patterns, which may not necessarily form a connected tree, a tree-structure probabilistic model was developed, called the probabilistic sibling-dependent tree Markov model, or PSTMM [36],[37]. This method not only included dependencies between parent and child, as in the hidden tree Markov model (HTMM) [38], but also included dependencies between consecutive siblings. Efficient algorithms were accordingly developed for the estimation of parameters and for training the model. This model was later improved for computational complexity while also maintaining the same level of performance. In this new ordered tree Markov model (OTMM) [39], instead of incorporating dependencies to both elder sibling and parent from each node, only one dependency was used, where the eldest sibling depended only on the parent, and each younger sibling only depended on its older sibling.

In order to retrieve the learned patterns directly from the model, a profile version of these models, called ProfilePSTMM, was subsequently developed to add insertion and deletion states in addition to the original match state. This model was tested on binding affinity data of galectins, which are known to recognize galactose residues, but had not been analyzed for longer patterns. In this experiment, a dimer structure was found to appear highly in the data, which was corroborated by experimental results [40].

## Conclusion

This tutorial briefly described several different bioinformatic methods for glycome research. With the further development of data resources and standards for data exchange, we hope that even better and newer methods to help understand the functioning of the glycome can be developed.
